# Laminin-Modified Dental Pulp Extracellular Matrix for Dental Pulp Regeneration

**DOI:** 10.3389/fbioe.2020.595096

**Published:** 2021-01-13

**Authors:** Jiahui Fu, Jianfeng Chen, Wenjun Li, Xiaomin Yang, Jingyan Yang, Huixin Quan, Haitao Huang, Gang Chen

**Affiliations:** ^1^Department of Stomatology, First Affiliated Hospital, Dalian Medical University, Dalian, China; ^2^Department of Oral Pathology, College of Stomatology, Dalian Medical University, Dalian, China

**Keywords:** laminin, dental pulp, extracellular matrix, odontoblast, regeneration

## Abstract

Native dental pulp extracellular matrix (DPEM) has proven to be an effective biomaterial for dental pulp regeneration. However, as a significant extracellular matrix glycoprotein, partial laminins were lost during the decellularization process, which were essential for odontoblast differentiation. Thereby, this study investigated the feasibility of LN supplementation to improve the surface of DPEM for odontoblast layer regeneration. The influences of laminin on cell adhesion and odontogenic differentiation were evaluated in vitro. Then, we fabricated laminin-modified DPEM based on the physical coating strategy and observed the location and persistency of laminin coating by immunofluorescent staining. Finally, laminin-modified DPEM combined with treated dentin matrix (TDM) was transplanted in orthotopic jaw bone of beagles (*n* = 3) to assess the effect of LNs on dental pulp tissue regeneration. The in vitro results showed that laminins could improve the adhesion of dental pulp stem cells (DPSCs) and promoted DPSCs toward odontogenic differentiation. Continuous odontoblastic layer-like structure was observed in laminin-modified DPEM group, expressing the markers for odontoblastogenesis, dentine matrix protein-1 (DMP-1) and dentin sialophosphoprotein (DSPP). Overall, these studies demonstrate that the supplementation of laminins to DPEM contributes to the odontogenic differentiation of cells and to the formation of odontoblast layer in dental pulp regeneration.

## Introduction

Dental pulp is a loose connective tissue containing vasculature and nerves as well as some specific cells, such as odontoblasts, to ensure the physiological properties of the dentine (Xuan et al., 2018). However, bacteria sometimes invades the pulp tissue to cause pulpitis, pulp necrosis, and subsequent apical periodontitis, which are commonly treated by removing the pulp and replacing it with inorganic materials via root canal treatment (RCT) (Eramo et al., 2018). This traditional treatment does not restore the biological function of the pulp.

Over the past 30 years, tissue-engineered approaches have been recognized as a promising future treatment model for achieving pulp healing and regeneration (Kawashima and Okiji, 2016).

To date, there is compelling evidence that native extracellular matrix (ECM) material could retain its inherent structural, biochemical, and biomechanical cues of tissues or organs (Hynes, 2009; Martino et al., 2014) to support the stem cell survival and proliferation, and specifically to guide stem cell differentiation (Uygun et al., 2010; Gilpin and Ott, 2015). Based on the technology of decellularization, native dental pulp extracellular matrix (DPEM) has also been fabricated and utilized as scaffold for dental pulp regeneration (Chen et al., 2015a; Hu et al., 2017).

The method used to prepare DPEM is the joint application of sodium dodecyl sulfate (SDS) and non-ionic surfactant Triton X-100 (Chen et al., 2015a), two chemicals that have presented many benefits in removing unwanted native constituents of the tissue (Gilpin and Yang, 2017). This strategy has so far been applied in the decellularization of whole organs (Ott et al., 2008; Uygun et al., 2010) and tissues (Sutherland et al., 2015; Paduano et al., 2017; Zhang et al., 2017). However, SDS is one ionic surfactant, and its application can cause damage to the structural and signaling proteins of ECM. For instance, the collagen in SDS-treated heart valves becomes compacted (Zhou et al., 2010), and the decellularized ECM of human and porcine lungs appears more fibrous than the structure of the smooth native tissue (O’Neill et al., 2013). Songlin Wang et al. show that the partial protein expression is decreased in the acellular pulp due to the decellularizing process (Hu et al., 2017). In our previous report, laminins located in the odontoblast layer were also lost accompanied by the process of decellularization (Chen et al., 2015a).

Laminins, as a key structural and biologically active component of basement membranes (BMs), could promote dentin formation and regulate odontoblast differentiation in tooth development (Yuasa et al., 2004; Tang and Saito, 2018), which are large, heterotrimeric, multidomain proteins constituted by the assembly of three disulfide-linked polypeptides, the α, β, and γ chains (Aumailley, 2013). During organogenesis, laminins could interact with many ECM molecules and bind cells through cell surface receptors, including integrins, syndecans, and α-dystroglycan, modulating cellular phenotypes and differentiation (Yap et al., 2019).

In the light of this consideration, the loss of laminins may affect the formation of the odontoblast layer and thus the effect of pulp regeneration. Therefore, we speculate that the supplementation of laminin in DPEM may contribute to regenerate a complete and continuous odontoblast layer. We have designed laminin-modified DPEM for dental pulp regeneration in the present study and have determined the feasibility for the repair of the odontoblast layer using laminin for a biofunctional scaffold coating.

## Materials and Methods

### Experimental Design

**FIGURE 1:**
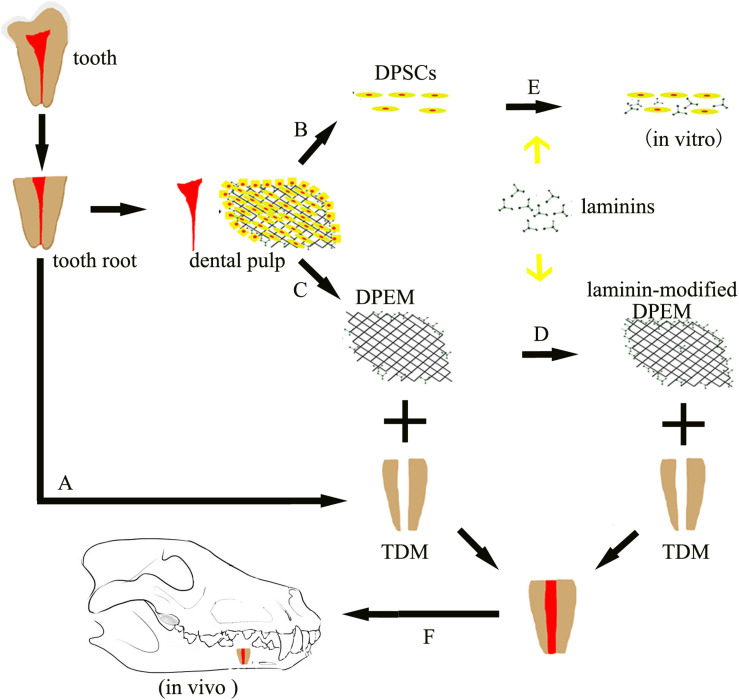
Schematic of the experimental design. Dental pulp extracellular matrix (DPEM) obtained from dental pulp tissues was fabricated into laminin-modified DPEM in this study. Treated dentine matrix (TDM) harvested from extracted teeth were used to simulate the dental pulp cavity. Then, laminin-modified DPEM as well as DPEM combined with TDM were transplanted into fresh premolar alveolar sockets for dental pulp regeneration. Meanwhile, dental pulp stem cells (DPSCs) were also isolated and cultured for *in vitro* experiments. A, TDM fabrication; B, cell culture; C, decellularization; D, laminin coating; E, DPSCs co-cultured with laminins *in vitro*; F, orthotopic transplantation; DPSCs, dental pulp stem cells; TDM, treated dentine matrix; DPEM, dental pulp extracellular matrix.

The overview of the experimental design can be obtained in Figure 1. DPEM was fabricated from the dental pulp tissues of swine. Laminins from Engelbreth–Holm swarm murine sarcoma BM were used to coat the surface of DPEM. In order to observe dental pulp regeneration *in vivo*, the premolars were first extracted from beagles and fabricated into treated dentine matrix (TDM), according to a published process (Li et al., 2011). Then, DPEM and laminin-modified DPEM scaffolds were, respectively, placed into root-shaped TDM, and the composites were transplanted into the jaw of beagles for dental pulp regeneration.

In this study, one implant was implanted on the left and right sides of each dog’s mandible, and three beagles were used that were obtained from the Animal Research Centre of Dalian Medical University (Dalian, China). The treatments and the study protocol complied with all committee regulations approved by the Ethics Committee for Animal Experimentation of the Dalian Medical University.

### Isolation and Culture of Dental Pulp Stem Cells (DPSCs)

#### DPSCs Culture

Human DPSCs were obtained from healthy wisdom teeth extracted for clinical purposes. Concretely, the teeth were transversely cut; then, the pulp tissues were aseptically collected from the dental pulp cavity. The pulp tissue was cut into small pieces and used for primary culture in Dulbecco’s modified Eagle’s medium (α-MEM, Hyclone, United States) supplemented with 10% fetal bovine serum (FBS, Hyclone, United States) in a humidified atmosphere at 37°C and 5% CO_2_, following the procedure reported by Shigeta et al. (Takizawa et al., 2019). Cell culture medium was refreshed every 2 days. DPSCs from passages 3–6 were used in the experiments.

#### Immunofluorescent (IF) Analysis

To describe the characterization of the obtained DPSCs, mouse primary antibodies against Vimentin (1:200 dilution, Abcam, United States) and CK-14 (1:200 dilution, Abcam, United States) were used in IF staining. Briefly, cell samples were fixed in 10% formalin for 2 h, rinsed in PBS for 15 min and then 0.2% Triton X-100 for 30 min. Samples were then blocked with 1% albumin bovine serum (BSA, Solarbio, China) for 1 h at room temperature. Primary antibodies were applied in blocking buffer overnight at 4°C, followed by secondary antibody (Alexa Fluor 488/555 Goat anti Mouse) (Invitrogen, Carlsbad, CA, United States) at 1:500 dilution for 2 h at 37°C. Samples were counterstained using DAPI (1:1000 dilution, Sigma, United States) and images acquired using inverted fluorescent microscope (Leica Optical, Germany).

### Influences of Laminins on DPSCs

#### Cell Adhesion

To observe the effect of laminins (1 mg/mL, L2020, Sigma, St. Louis, MO, United States) on cell adhesion, various concentrations of laminins were used in this study. Concretely, laminins were serially diluted in ultrapure distilled water to 0.1, 1, 10, and 100 μg/mL. Then, various concentrations of laminins were poured into enzyme-free sterile glass culture dishes (Supin, Nantong, China) and placed in an aseptic incubator with a humidified atmosphere at 37°C and 5% CO_2_ for 2 days until the water was dried. The laminin-coated Petri dishes were obtained and seeded with DPSCs at a density of 6 × 10^4^/mL (culture media: 3 mL/well) in α-MEM supplemented with 10% FBS. A non-coated Petri dish was used as control in this experiment. Photographs of cells were taken using a phase contrast microscope (Olympus, Tokyo, Japan) at 1 and 24 h, respectively. To compare the number of cell adhesions at 24 h, three images were randomly selected and analyzed by ImageJ software (National Institutes of Health, Bethesda, MD, United States) for each group.

#### Odontogenic Differentiation

To analyze the influences of laminins on cells, real-time quantitative polymerase chain reaction (RT-qPCR) analysis was used in this study. Briefly, laminin solution was first diluted in culture medium to 100 μg/mL as experimental medium. Then, 1 × 10^4^ DPSCs were seeded and cultured in culture medium containing laminin or not. After 1 week of cell culture, total RNA was isolated using RNAiso Plus (TaKaRa, Japan) according to the manufacturer’s instructions, followed by complementary DNA (cDNA) synthesis and PCR procedures. The cycling conditions were as follows: 95°C for 10 min, 45 cycles at 95°C (15 s each), and 60°C for 1 min. The data was analyzed by the 2^–ΔΔCt^ method (Livak and Schmittgen, 2001). The primer sequences for analyzed genes are listed in Table 1.

**TABLE 1:** Oligonucleotide primer sequences.

Target cDNA	Primer sequence (5′-3′)	Product size (bp)	NCBI no.
GAPDH	F CCATCTTCCAGGAGCGAGATC R CCCCAGCCTTCTCCATGGT	105	NM_001289746
DLX-5	F CAACCAGCCAGAGAAAGAAGTGA R GGCAAGGCGAGGTACTGAGT	118	NM_005221.6
SP7	F AGGCCCTTCGTCTGCAACT R GGTGCGCTGGTGTTTGCT	117	NM_001173467.3
COL-2	F GAAGAACTGGTGGAGCAGCAA R GTGGACAGCAGGCGTAGGAA	119	NM_001844.4
COL-10	F TGGGTAGGCCTGTATAAGAATGG R CCATTTGACTCGGCATTGG	117	NM_000493.4
MMP13	F GGACCCTGGAGCACTCATGT R CATTTGTCTGGCGTTTTTGGA	119	NM_002427.3
DSPP	F AGAAGACAATGGCAGCCAAAG R CTGCTGGGACCCTTGATTTC	116	NM_014208.3
ALP	F AATGCCTGGATCCTGTTGACA R TGCCCATGCAACACTTCAAG	118	NM_003064.4
RUNX2	F CGTGGCCTTCAAGGTGGTA R AATCTCAGATCGTTGAACCTTGCT	117	NM_001015051.3
MSX2	F CCGCCGCCAAGACATATG R TGCACGCTCTGCAATGGA	120	NM_002449.5

### Fabrication and Biocompatibilities of Laminin-Modified DPEM

#### DPEM Fabrication

To fabricate DPEM, the first and second incisor teeth, second and third molar tooth germs (M2 and M3, respectively) were obtained from the recently discarded swine jaws as in our previous report (Chen et al., 2015a). Dental pulp tissues were peeled off from the obtained tooth and rinsed thoroughly in phosphate buffer solution (PBS, Solarbio, China). Then, the prepared dental pulp tissues were treated according to a decellularization process (Ma et al., 2013). Concretely, the dental pulp tissues were incubated in 1% SDS (Bio-Rad, United States) for 12 h, followed by 30 min of 1% Triton X-100 (Sigma, United States) in deionized water. After decellularization, the sterilization of DPEM was performed by washing DPEM in PBS for 30 min, followed by immersing it in a solution containing 100 units/ml penicillin (Hyclone, United States) and 100 mg/ml streptomycin (Hyclone, United States) for 48 h (Ott et al., 2008).

#### Laminin-Modified DPEM Fabrication

To obtain the laminin-modified DPEM, laminin (1 mg/mL, L2020, Sigma, St. Louis, MO, United States) purified from the basement membrane of Engelbreth–Holm swarm murine sarcoma was diluted in ultra-pure distilled water to 100 μg/mL (Rajabi et al., 2018). Then, the fabricated DPEM (10 mm × 5 mm × 3 mm) was directly coated with 100 μg/mL laminin solutions for 2 h at 37°C. After that, laminin-modified DPEM were prepared for use in this study.

#### IF Analysis

To detect the cell nucleuses in DPEM, 2-(4-Amidinophenyl)-6-indolecarbamidine dihydrochloride (DAPI) (1:1000 dilution, Sigma, United States) staining was used. The samples were prepared according to the previous report (Chen et al., 2015a). Tissue sections (5 μm) were mounted using DAPI-containing mounting media for 3 min and observed by inverted fluorescent microscope (Leica Optical, Germany).

To observe the distribution of laminins, the tissue sections of DP, DPEM, and laminin-modified DPEM were prepared to detect the expressions of laminin (1:250 dilution, Abcam, United States). After DPSCs were seeded on the surface of laminin-modified DPEM for 1 week, the tissue sections of laminin-modified DPEM/cells were prepared to test the persistency of laminins. IF analysis was used in these two experiments.

#### Scanning Electron Microscope (SEM) Analysis

To observe the effects of laminin on cell proliferation, SEM was used to observe the cells on DPEM and laminin-modified DPEM. Briefly, the same number of DPSCs were seeded on DPEM and laminin-modified DPEM for 1 week. DPEM and DP as control samples were prepared. Then, the four samples were prefixed by immersion in 2% glutaraldehyde in 0.1 M PBS, and postfixed for 2 h in 1% osmic acid dissolved in PBS. Samples were then treated in a graded series of ethanol and t-butyl alcohol, dried in a freeze dryer (ES-2030, Hitachi, Tokyo, Japan), platinum-coated using an ion coater (IB-3, Eiko, Japan), and observed under SEM (ZEISS, Germany).

### *In situ* Jawbone Transplantation in Beagle Dogs

All animal experiments described in this study were conducted in accordance with protocols approved by the Ethics Committee of Dalian Medical University. The beagles (8–12 kg) were purchased from the Laboratory Animal Center of Dalian Medical University (Dalian, China).

#### Preparation of Dental Pulp Cavity

To simulate the morphology of the dental pulp cavity, the TDM were fabricated and used in the dental pulp cavity for dental pulp regeneration in this study. Briefly, we harvested the extracted teeth from beagles and then removed the crown, dental pulp, predentin, and cementum using a mechanical method (Li et al., 2011). Finally, the dentin matrix was treated with 17% ethylenediaminetetraacetic acid (EDTA, Sigma, United States) for 12 min, 10% EDTA for 12 min, and 5% EDTA for 20 min. For sterilization, TDM was immersed in sterile PBS supplemented with 100 units/ml penicillin and 100 mg/ml streptomycin for 24 h in 37°C, then washed with sterile deionized water for 10 min in an ultrasonic cleaner and finally stored in α-MEM at 4°C.

#### Transplantation

Three beagles had their second premolars removed before transplantation. ECM materials (DPEM, laminin-modified DPEM, *n* = 3) combined with TDM as composites for dental pulp regeneration were prepared and transplanted into the extraction wound of the second premolar. The surgical procedure was performed under deep anesthesia.

#### CBCT Analysis and Histology Analysis

Beagles were sacrificed after 12 weeks, and the mandibles were harvested and fixed with 4% paraformaldehyde. Cone-beam computed tomography (CBCT, Vatech, South Korea) was used to observe the harvested samples. All samples were then demineralized for 1 month, embedded, and sectioned into 5-μm-thick sections for histological analyses according to the manufacturer’s recommended protocols. As mentioned earlier, the prepared sections (5 μm) were also stained with H&E stain. For immunohistochemistry analysis, the primary antibodies used in this study were as follows: rabbit anti-DSPP (1:100 dilution; Abcam, MA, United States), rabbit anti-collagen type I (Col-1, 1:200 dilution, Bioss, Inc., Woburn, MA, United States), rabbit anti-laminin (1:50 dilution, Abcam, MA, United States), rabbit anti-DMP-1 (1:50 dilution, Invitrogen, Eugene, OR, United States).

## Results

### Isolation and Culture of DPSCs

**FIGURE 2:**
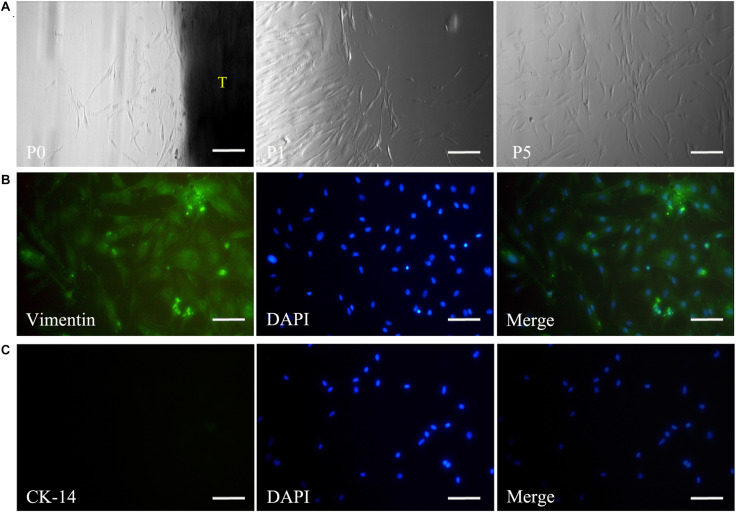
Isolation and culture of DPSCs. The panel **(A)** shows that the harvested DPSCs with the typical spindle morphology of mesenchymal cells could maintain a good state of proliferation from passage 0 to 5. Immunofluorescence staining reveals vimentin expression **(B)**, but negative staining for CK-14 in DPSCs **(C)**. Scale bars: 200 μm. P0, passage 0; P1, passage 1; P5, passage 5; T, tissue blocks.

Primary DPSCs were successfully obtained from the dental pulp of the impacted tooth as in our previous reports (Chen et al., 2015a). From passage 1 (P1) to 5 (P5), the cells kept the typical spindle morphology of mesenchymal cells (Figure 2A) and maintained a good state of proliferation. Meanwhile, IF staining revealed that the cells were positive for mesenchymal cell marker vimentin (Figure 2B) and negative for epithelial cell marker CK-14 (Figure 2C).

### Influences of Laminins on DPSCs

#### Cell Adhesion

**FIGURE 3:**
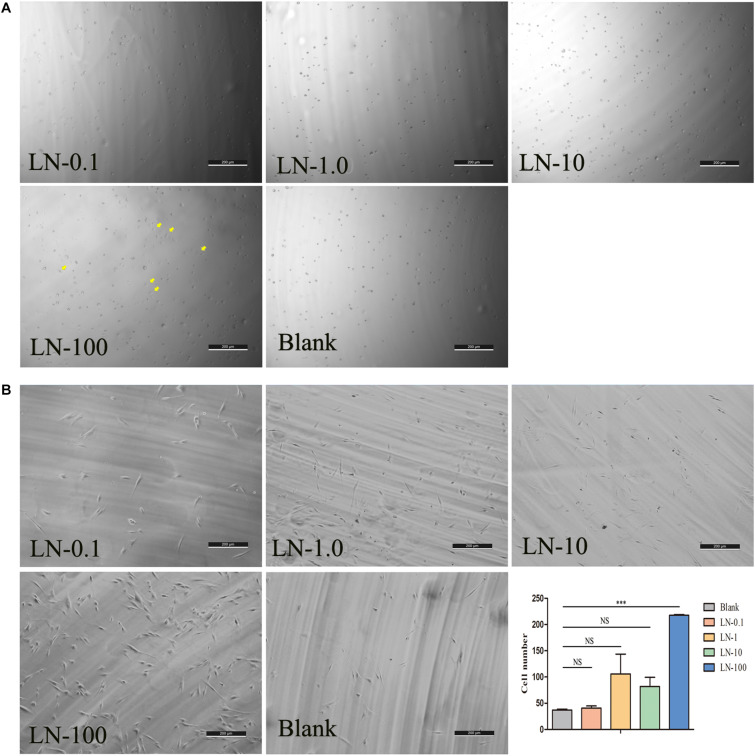
The effects of LN on cell adhesion. The panel shows the representative photomicrographs of cells at 1 **(A)** and 24 h **(B)** postinoculation. LN was coated on plates at concentrations of 0.1 (LN-0.1), 1 (LN-1), 10 (LN-10), and 100 μg/mL (LN-100). At 24 h, the number of cell adhesions in different concentration groups was counted by ImageJ software. LN, laminin; (Yellow arrows labeled the attached cells at 1 h).

To investigate the effects of laminins on cell adhesion, laminins were coated on plates at concentrations of 0.1 (laminin-0.1), 1 (laminin-1), 10 (laminin-10), and 100 μg/mL (laminin-100). The results showed that most cells in all the groups remained spherical in shape. However, we found that some cells started attaching and forming protrusions in the laminin-100 group at 1 h (Figure 3A). After being co-cultured for 24 h, the adherent cells were spindle-shaped and flat in all groups (Figure 3B), whereas the number of cells in the laminin-100 group was the highest in all groups. The histogram showed that the number in the laminin-100 group was statistically higher than the blank group, which demonstrated laminins at the concentration of 100 μg/mL could promote cell adhesion significantly (Tang and Saito, 2018).

#### Odontogenic Differentiation

**FIGURE 4:**
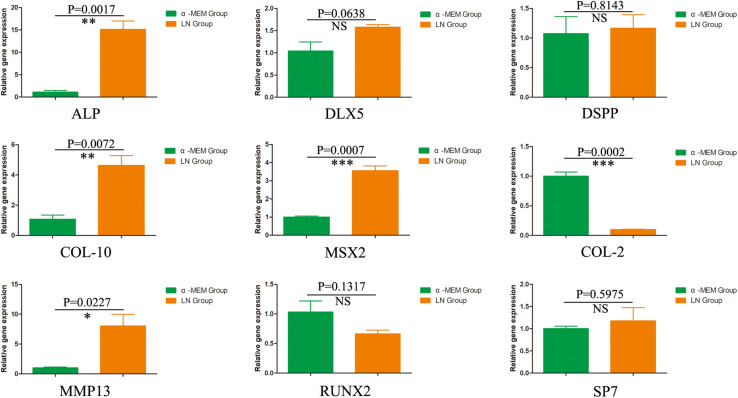
The odontogenic influences of laminin on DPSCs. After 1 week, DPSCs co-cultured with laminins at the concentration of 100 μg/mL upregulated the early odontogenic markers, ALP, MSX2, Col-10, and MMP13. However, laminins did not change the expression of other osteogenic/odontogenic markers, such as DLX5, DSPP, RUNX2, and SP7. Meanwhile, laminins downregulated the expression of cartilage-specific gene Col-2. ^∗^*p* < 0.05, ^∗∗^*p* < 0.01, ^∗∗∗^*p* < 0.001, NS means no significance.

To investigate the odontogenic influences of laminins on DPSCs, DPSCs were co-cultured with laminins at a concentration of 100 μg/mL for 1 week. Real-time PCR revealed increased expression of relative quantification to the early odontogenic genes, alkaline phosphatase (ALP) (nearly 13.5-fold higher than the control group), muscle segment homeodomain homeobox 2 (MSX2) (3.5-fold), collagen type X alpha 1 (COL-10) (4.3-fold), and matrix metallopeptidase 13 (MMP13) (7.9-fold). The expression of other osteogenic/odontogenic markers, such as distal-less homeobox 5 (DLX5), dentin sialophosphoprotein (DSPP), Runt-related transcription factor 2 (RUNX2), and Sp7 transcription factor (SP7), did not change significantly. Meanwhile, laminins downregulated the expression of cartilage-specific gene collagen type II alpha 1 (COL-2) (minus 10.0-fold) (Figure 4).

### Characterization of Laminin-Modified DPEM

#### DPEM

**FIGURE 5:**
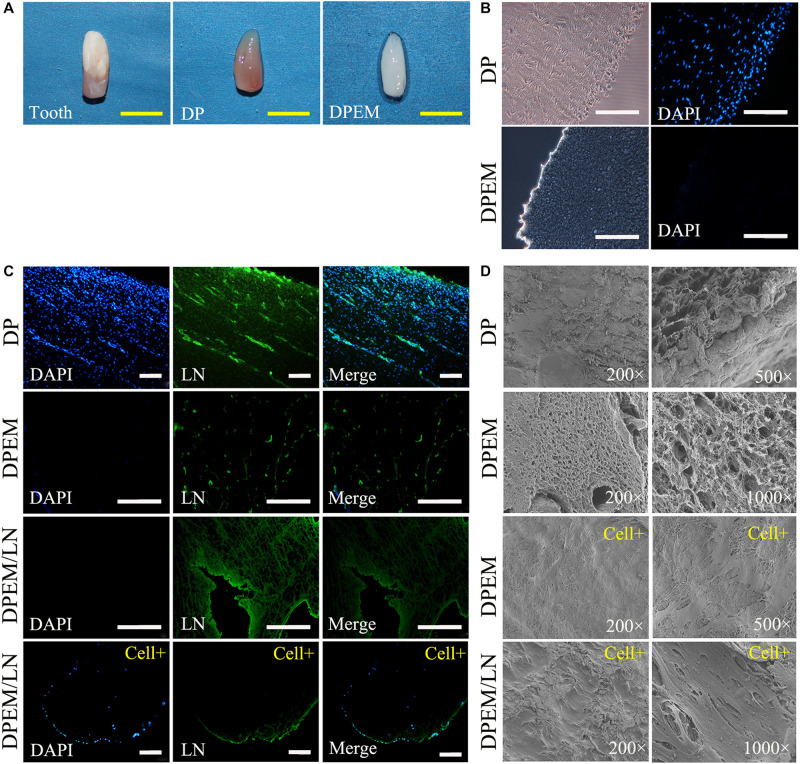
Fabrication and characterization of laminin-modified DPEM. The panel **(A)** shows the obtained developing tooth from swine with rich dental pulp tissues (DP). After the process of decellularization, the fabricated DPEM preserved the predetermined size and shape of dental pulp tissues. The cells in DP were not visualized in DPEM **(B)** by staining nuclei with the fluorescent dye DAPI. The panel **(C)** shows a continuous, brightly green fluorescence (LN-bound Alexa 488) along the surface of LN-coated graft but not on the surface of DPEM. Meanwhile, after being seeded with DPSCs for 1 week, DPEM/LN shows the persistency of LN coating though the intensity was visually reduced. For further observation, SEM analysis **(D)** was used to observe the microstructure of DP and DPEM. After cell seeding for 1 week, the SEM images of laminin-modified DPEM (DPEM/LN) showed better cell viability than DPEM. DPEM, dental pulp extracellular matrix; DPEM/LN, laminin-modified DPEM; DP, dental pulp; Yellow scale bars: 1 cm; White scale bars: 200 μm.

The obtained developing tooth from swine with rich, highly vascularized, reddish-colored, dental pulp tissues (Figure 5A). After decellularization, the fabricated DPEM appeared white as compared to DP but preserved the predetermined size and shape of dental pulp tissues. Meanwhile, DAPI staining showed the absence of cell nuclei in DPEM specimens, indicating complete decellularization (Figure 5B) as compared to native DP.

#### Laminin Coating Persistency

The expression patterns of laminin in DP, DPEM, and DPEM/LN materials were tested utilizing IF staining (Figure 5C). The DP presented strong fluorescent signals in the BM of vascular structures and the odontoblast layer (Figure 5C: DP). After decellularization, laminins at the BM of vascular structures were preserved but lost at the place of the odontoblast layer in DPEM (Figure 5C: DPEM), consistent with the published reports (Chen et al., 2015a). Alexa Fluor 488-coupled laminin coating, a continuous, bright green fluorescence, was presented along the surface of laminin-modified DPEM (Figure 5C: DPEM/LN), meaning the method of laminin coating referred to previous reports (Toshmatova et al., 2019) would be feasible to the fabrication of laminin-modified DPEM.

When DPSCs were seeded on laminin-coated DPEM for 1 week, the fluorescence still could be detected though the intensity was visually reduced (Figure 5C: DPEM/LN/cell +).

#### SEM Analysis

Scanning electron microscope images demonstrated that the microstructure of dental pulp tissues possessed a dense surface (Figure 5D: DP), but the decellularized dental pulp tissues (Figure 5D: DPEM) presented a loose and porous structure. After cell seeding for 1 week, the SEM images of laminin-coated DPEM showed better cell viability than DPEM (Figure 5D: DPEM/Cells). The cells presented multilayer growth on the surface of the laminin-coated DPEM (Figure 5D: DPEM/LN/cell +).

### *In vivo* Dental Pulp Regeneration Induced by Laminin-Modified DPEM in Beagle Dogs

**FIGURE 6:**
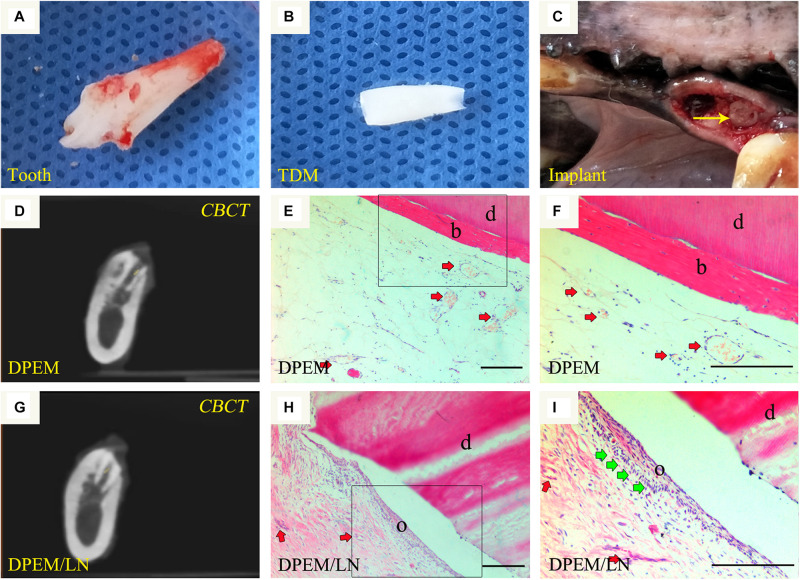
Orthotopic implantation for dental pulp regeneration. The extracted tooth **(A)** from beagles were fabricated into treated dentin matrix (TDM) **(B)**, which could provide a large enough pulp cavity for dental pulp regeneration. Then, DPEM/TDM and DPEM/LN/TDM were separately implanted into alveolar sockets **(C)**. After 12 weeks, CBCT showed the harvested samples at the implant sites **(D,G)**. H&E staining verified that the cellular cementum/bone-like structures were regenerated obviously in the DPEM group **(E,F)**. In the DPEM/LN group, odontoblastic layer–like structures were generated on the interface between dental pulp–like tissues and dentin matrix **(H,I)**. (The yellow arrow labels the implant; the red arrows label the generated vessels in dental pulp tissues and the green arrows label the odontoblastic layer). TDM, treated dentine matrix; DPEM, dental pulp extracellular matrix; DPEM/LN, laminin-modified DPEM; DP, dental pulp. d, dentin matrix; b, bone-like tissues; o, odontoblastic layer. Scale bars: 200 μm.

The beagle teeth could be completely extracted (Figure 6A). Then, the intact tooth roots were fabricated into TDM (Figure 6B), which could provide a large enough pulp cavity to simulate a clinical immature root for dental pulp regeneration as in our previous report (Chen et al., 2015a). Then, DPEM/TDM and DPEM/LN/TDM composites were separately implanted into alveolar sockets (Figure 6C). After 12 weeks, CBCT images showed the harvested implants without obvious immune rejection and severe inflammatory reaction (Figures 6D,G), indicating the good biocompatibility of implant biomaterials. H&E staining verified that the cellular cementum/bone-like structures and blood vessels were regenerated obviously in the DPEM-alone group (Figures 6E,F). Thus, DPEM could be revascularized effectively to nourish the dentine tissues (Chen et al., 2015a). In the DPEM/LN group, odontoblastic layer-like structures were observed on the interface between dental pulp–like tissues and the dentin matrix (Figures 6H,I).

**FIGURE 7:**
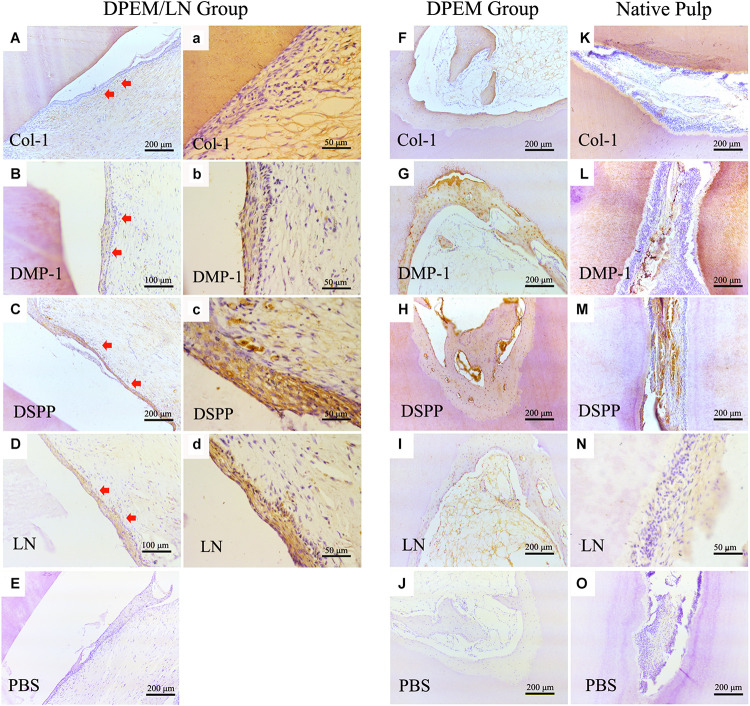
IHC examination of the composites for dental pulp regeneration *in vivo*. The generated odontoblastic layer–like structures in DPEM/LN group were positive for the related odontogenic markers, Col-1 **(A,a)**, DMP-1 **(B,b)**, DSPP **(C,c)**, and LN **(D,d)**. The expression patterns of native dental pulp tissues are shown in panels **(K–N)**. In DPEM group, the regenerated cellular cementum/bone-like structures are observed in **(F–I)** without the odontoblastic layer–like structure. As negative control, the harvested samples were negative for PBS **(E,J,O)**. DPEM/LN, laminin-modified DPEM; LN, laminin; Col-1, collagen-1; DMP-1, dentin matrix protein-1; DSPP, dentin sialophosphoprotein; PBS, phosphate buffer solution; (Red arrows label the generated odontoblastic layer–like structure).

Immunohistochemistry was used to evaluate the regenerated pulp-like tissue (Figure 7). The results showed that the generated odontoblastic layer–like structures in DPEM/LN group were positive for the related odontogenic markers, Col-1 (Figures 7A,a), DMP-1 (Figures 7B,b), DSPP (Figures 7C,c), and LN (Figures 7D,d), which were similar to the expression patterns of native dental pulp tissues (Figures 7K–N). The harvested DPEM group regenerated the cellular cementum/bone-like structures (Figures 7F–I). As a negative control, the harvested samples were negative for PBS (Figures 7E,J,O).

## Discussion

In the present study, we describe the amelioration of DPEM by incorporation of laminins, aiming at the formation of a continuous odontoblastic layer in dental pulp regeneration. DPEM as a unique and effective scaffold has been obtained and utilized for dental pulp regeneration (Zhang et al., 2017). However, associated with decellularization, one main challenge is that partial ECM components are often stripped away or altered by the detergents used to remove cellular debris (Young et al., 2019). Thus, the loss of ECM components may affect the effect of DPEM on dental pulp regeneration.

Laminins, as effective biological molecules, were lost partially in fabricated DPEM (Chen et al., 2015a), which could control odontoblast proliferation and differentiation through engaging several integrin receptors of cell surfaces or growth factors (Tang and Saito, 2018). In *in vitro* culture, there were the highest number of adherent DPSCs in the laminin-100 group (Figure 3B). It demonstrated that 100 μg/mL laminins could contribute to mesenchymal cell adhesion in dental implants (Min et al., 2013). Actually, except for promoting the cell adhesion, the influence of laminins on the osteo/odontoblastic differentiation was also evaluated in this study. The expressions of related odontogenic genes were assessed by RT-PCR in this study (Figure 4). The results showed that ALP was upregulated nearly 13.5-fold higher than the control group (Figure 4), which is an early marker of odonto/osteoblast differentiation. The previous report demonstrates that laminins could activate the ALP activity of an odontoblast-like cell line (Tang and Saito, 2018). Thus, the enhancement of ALP in the present study denotes that laminins can promote the DPSC differentiation process toward mineralizing tissue (Tang and Saito, 2017). Meanwhile, Msx2 homeoprotein, a key transcription factor of dental and periodontal tissue formation (Fujii et al., 2015), was also upregulated about 3.5-fold higher than control group (Figure 4). In a previous report, Towler DA et al. detected the expression of Msx2 in ovoid pre-odontoblasts (Bidder et al., 1998). It is involved in many molecular pathways controlling mineralized tissue homeostasis, such as the Wnt/sclerostin pathway (Korah et al., 2019). In our study, the expression of Col-10 was upregulated nearly 4.3-fold (Figure 4). During the stages of ECM protein secretion and mineralization, Col-10 genes are expressed in mesenchymal cells of developing teeth and are involved in odontogenic differentiation (Debiais-Thibaud et al., 2019). MMP-13 is a member of the zinc-dependent endopeptidases playing an important role during ECM formation. The expression of MMP-13 in DPSCs has been upregulated nearly 7.9-fold in the laminin group (Figure 4). It has been demonstrated that mature human odontoblasts could express MMP-13 (Nurmenniemi et al., 2010). The MMP-13 expression is mediated via activation of the ERK and NF-κB signal pathways in odontoblasts, involved in remodeling of the pulp-dentine complex (Zhang et al., 2013).

Meanwhile, for the four genes, DLX5, DSPP, RUNX2, and SP7, there was no significant change of expression in the laminin group compared with control (Figure 5). RUNX2, as one specific osteoblast transcription factor, was slightly decreased in this study. COL-2, as one cartilage-specific gene, was inhibited significantly (Figure 5). The expression of both RUNX2 and COL-2 suggest commitment into an odontoblastic phenotype as opposed to osteoblastic (Farshdousti Hagh et al., 2015) or chondroblastic phenotype (Agrawal and Pramanik, 2019). DLX5, as one non-specific osteoblast transcription factor (Lee et al., 2013), can form a heterodimer with Mxs2 regulating common target genes, which includes osteocalcin, bone sialoprotein, osteopontin, and collagen type I alpha1 (Farshdousti Hagh et al., 2015). DSPP was slightly increased in this study, which was a typical factor in odontoblasts of the pulp-dentin complex (Guo et al., 2019). Sp7, a zinc finger transcription factor, could coordinately modulate RUNX2 and DLX5 proteins at levels appropriate for optimal osteo/odontoblastic differentiation and function (Barone et al., 2014). Based on the slight increment of DSPP and Sp7 expression, we speculate that additional mineralized microenvironments may be required in *in vitro* culture (Yuasa et al., 2004; Tang et al., 2018).

Diverse methods, such as physical coating, covalent chemical bonding, and blended electrospinning have been employed to immobilize laminin protein on the surfaces of substrates (Koh et al., 2008). In the present study, we fabricated laminin-modified DPEM based on the physical coating strategy (Figure 5). The fluorescence of laminin still could be detected on the surface of the scaffold even after being seeded and cultured *in vitro* for 1 week (Figure 5C), showing the good persistence of laminin coating. Payam Akhyari et al. demonstrated the physical coating of laminin could persist on decellularized grafts for at least 8 weeks *in vivo* (Toshmatova et al., 2019). Meanwhile, SEM results showed DPSCs presented multilayer growth on the surface of the laminin-coated DPEM (Figure 5D). Therefore, the above results demonstrated that the physical coating of laminin could functionalize the DPEM surface to improve the adhesion of DPSCs. This observation is in accordance with the reported result that the laminin-coated surface could provide the functional biochemical signals for enhancing cell attachment and proliferation (Nie et al., 2018).

The *in vivo* transplantation is the “gold standard” to verify the validity of one strategy for tissue regeneration. Cell transplantation (Zhu et al., 2018) and cell homing (Kim et al., 2010), as two strategies, have been applied in dental pulp regeneration. The difference between the two strategies is the requirement of exogenous stem cells as seed cells. The cell homing strategy without the requirement for exogenous stem cells may avoid difficulties in obtaining regulatory approval, stem cell isolation and processing, the relatively high cost associated with cell cryopreservation, expansion, and a biological risk of immune-rejection, infection, and tumorigenesis (Kim et al., 2013). Thus, in this study, laminin-modified DPEM combined with TDM–one cell-free strategy was investigated for dental pulp regeneration *in vivo*.

Whatever the pulp-regeneration strategy, the regenerated tissues in root canals after the application of clinical regeneration protocols seem to be of periodontal origin as evinced by the presence of periodontal ligament-like, cementum-like, bone-like tissues (Kim, 2017). Laminin-modified DPEM combined with TDM (Figures 6A,B) were transplanted into the mandibles of beagles for 12 weeks. Odontoblastic layer–like structures were observed on the interface between dental pulp–like tissues and dentin matrix (Figures 6H,I) in the DPEM/laminin group. In contrast, the cellular cementum/bone-like structure was regenerated obviously in the DPEM-alone group (Figures 6E,F). Thus, the laminin coating may be useful to provide the support and functional biochemical signals for diverting the cellular events toward pulp-dentin complex regeneration rather than cementum-like tissue repair (Kim, 2017).

Meanwhile, DSPP and DMP-1 are the two most frequently used markers for odontoblastogenesis (Chen et al., 2015b), and laminins are widely expressed in tooth BM (Yuasa et al., 2004). DSPP, DMP-1 as well as laminin were positive in the generated odontoblastic layer–like structures verifying the generation of the odontoblastic layer (Figure 7). However, laminins showed limited capacity in inducing DSPP expression in *in vitro* culture (Figure 5; Tang et al., 2018), but DSPP was positive in *in vivo* results (Figure 7). The possible explanation is that a single growth factor cannot construct a complete odontogenic microenvironment, which requires the participation of laminin, DPEM, and TDM.

## Conclusion

In conclusion, we successfully fabricated laminin-modified DPEM and illustrated the roles of laminins in odontogenic differentiation *in vitro*. Meanwhile, the present study shows the effects of laminins on the generation of an odontoblast layer and provides a feasible strategy for the regeneration of dental pulp tissues. However, it is necessary to further explore an appropriate preservation for laminin-modified DPEM to facilitate clinical use.

## Data Availability Statement

The datasets presented in this study can be found in online repositories. The names of the repository/repositories and accession number(s) can be found below: https://www.ncbi.nlm.nih.gov/, NM_001289746; https://www.ncbi.nlm.nih.gov/, NM_005221.6; https://www.ncbi.nlm.nih.gov/, NM_001173467.3; https://www.ncbi.nlm.nih.gov/, NM_001844.4; https://www.ncbi.nlm.nih.gov/, NM_000493.4; https://www.ncbi.nlm.nih.gov/, NM_0024 27.3; https://www.ncbi.nlm.nih.gov/, NM_014208.3; https://www.ncbi.nlm.nih.gov/, NM_003064.4; https://www.ncbi.nlm.nih.gov/, NM_001015051.3; https://www.ncbi.nlm.nih.gov/, NM_002449.5.

## Ethics Statement

The studies involving human participants were reviewed and approved by the Ethics Committee of the First Affiliated Hospital of Dalian Medical University. The patients/participants provided their written informed consent to participate in this study. The animal study was reviewed and approved by the Ethics Committee for Animal Experimentation of the Dalian Medical University.

## Author Contributions

GC: designing experiments and supervision. JF and JC: writing – original draft preparation and data curation. WL, XY, and HQ: animal surgery, scaffold preparation, and evaluation. JY: writing, reviewing, and editing. JF and WL: histological evaluation, cell isolation, and culture. All authors contributed to the article and approved the submitted version.

## Conflict of Interest

The authors declare that the research was conducted in the absence of any commercial or financial relationships that could be construed as a potential conflict of interest.
